# Impact of Plasma Epstein-Barr Virus-DNA and Tumor Volume on Prognosis of Locally Advanced Nasopharyngeal Carcinoma

**DOI:** 10.1155/2015/617949

**Published:** 2015-01-31

**Authors:** Meng Chen, Li Yin, Jing Wu, Jia-Jia Gu, Xue-Song Jiang, De-Jun Wang, Dan Zong, Chang Guo, Huan-Feng Zhu, Jian-Feng Wu, Xia He, Wen-Jie Guo

**Affiliations:** Department of Radiation Oncology, Jiangsu Cancer Hospital & Institute of Cancer Research, Nanjing Medical University, Nanjing 210009, China

## Abstract

This retrospective study aims to examine the association of plasma Epstein-Barr virus- (EBV-) DNA levels with the tumor volume and prognosis in patients with locally advanced nasopharyngeal carcinoma (NPC). A total of 165 patients with newly diagnosed locally advanced NPC were identified from September 2011 to July 2012. EBV-DNA was detected using fluorescence quantitative polymerase chain reaction (PCR) amplification. The tumor volume was calculated by the systematic summation method of computer software. The median copy number of plasma EBV-DNA before treatment was 3790 copies/mL. The median gross tumor volume of the primary nasopharyngeal tumor (GTVnx), the lymph node lesions (GTVnd), and the total GTV before treatment were 72.46, 23.26, and 106.25 cm^3^, respectively; the EBV-DNA levels were significantly correlated with the GTVnd and the total GTV (*P* < 0.01). The 2-year overall survival (OS) rates in patients with positive and negative pretreatment plasma EBV-DNA were 100% and 98.4% (*P* = 1.000), and the disease-free survival (DFS) rates were 94.4% and 80.8% (*P* = 0.044), respectively. These results indicate that high pretreatment plasma EBV-DNA levels in patients with locally advanced NPC are associated with the degree of lymph node metastasis, tumor burden, and poor prognosis.

## 1. Introduction

Nasopharyngeal carcinoma (NPC) is a malignant tumor with obvious ethnic aggregation and geographical differences and is one of the most common malignant tumors in southern China and southeast Asia. The incidence of NPC is mainly related to genetic and environmental factors [1, 2]. Currently, the 2014 National Comprehensive Cancer Network (NCCN) recommends radiotherapy (RT) alone for early-stage NPC. Concurrent chemoradiotherapy (CCRT) followed by adjuvant chemotherapy (AC) for locoregionally advanced NPC (Category 2A) and CCRT alone (Category 2B) or induction chemotherapy (Category 3) followed by CCRT are also options. Platinum-based combination chemotherapy are option for metastatic patients (Category 2A) (http://www.nccn.org/professionals/physician_gls/pdf/head-and-neck.pdf). Although great progress has been made in the clinical efficacy of NPC treatment in recent years, local recurrence or distant metastasis is still a major problem for patients with locally advanced NPC. It has been confirmed that the occurrence and development of NPC are closely related to Epstein-Barr virus (EBV) infection, and plasma EBV-DNA is considered an effective indicator in the diagnosis of patients with NPC [3]. In addition, studies have suggested that the primary tumor volume is an important indicator predicting the prognosis of patients with NPC, and the larger the tumor volume, the poorer the prognosis [4]. The treatment outcomes of patients with stages III and IVA/B NPC are poor, and the main reason for treatment failure is local recurrence or distant metastasis [5]. It is particularly important to study the prognostic factors affecting the survival of patients with NPC to guide the treatment. This study reviews 165 patients with locally advanced NPC receiving intensity-modulated radiation therapy (IMRT) combined with concurrent chemotherapy and determines whether there is a correlation between pretreatment plasma EBV-DNA levels and tumor volume as well as its predictive role in assessing prognosis.

## 2. Materials and Methods

### 2.1. Patient Information

A total of 165 patients with newly diagnosed locally advanced NPC were identified in our hospital from September 2011 to July 2012. Patient assessment before treatment included a detailed physical examination, electronic nasopharyngoscopy, unenhanced and enhanced MRI scans of the nasopharynx and whole neck, plain and enhanced CT scans of the chest and abdomen, whole body bone scintigraphy, complete blood and biochemical examinations, EBV-DNA copy number, and thyroid function. Enrolled patients fulfilled the following criteria: (1) pathologically confirmed NPC; (2) locally advanced NPC including stage III/IVa/IVb in accordance with the 2009 Union for International Cancer Control (UICC) staging system; (3) receiving IMRT with concurrent chemotherapy; and (4) complete clinical data.

Patient information is shown in Table 1. This study was approved by the hospital review board and was conducted in accordance with the ethical guidelines of the Declaration of Helsinki.

### 2.2. Therapeutic Strategies

#### 2.2.1. Radiotherapy

All patients received IMRT using a 6 MV linear accelerator (Varian Company, USA). Based on the pretreatment MRI findings, according to the definition in the ICRU no. 50 and no. 62 reports, the therapeutic target was delineated layer by layer on enhanced CT, and the GTVnx (primary tumor site and violation range visible on imaging and clinical examination) received a dosage of 70–76 Gy with a single dose of 2.10–2.25 Gy, and the GTVnd (cervical lymph nodes) received a dosage of 64–70 Gy with a single dose of 2.00–2.25 Gy. The defined dose for crisis organs was assessed for the plan requirements in accordance with the RTOG0615 [6] provisions.

#### 2.2.2. Chemotherapy

Patients received two cycles of concurrent chemotherapy with paclitaxel and nedaplatin during radiotherapy. Paclitaxel 135 mg/m² was administered on day 1 and nedaplatin 80 mg/m² on days 1–3, once every four weeks. Blood and biochemistry parameters in addition to EBV-DNA copy number were monitored.

### 2.3. Measurement Techniques

#### 2.3.1. Tumor Volume

After completing target delineation in all patients, GTVnx and GTVnd were automatically generated by the Varian Company TPS treatment planning system, followed by calculation of the total GTV.

#### 2.3.2. Evaluation of EBV-DNA Levels

Peripheral venous blood (3 mL) was collected before treatment from each patient into EDTA-containing tubes and centrifuged at 3000 rpm for 5 min. Total plasma DNA was extracted using a QIAamp DNA Blood Mini Kit (Qiagen, Hilden, Germany). Fluorescence polymerase chain reaction (PCR) was carried out using EBV PCR quantitative diagnostic kit (Da-An Genetic Diagnostic Center, Guangzhou, China). EBV-DNA levels were measured using real-time quantitative toward the* Bam*HI-W region of the EBV genome. The experimental data were analyzed using Applied Biosystems 7300 SDS software for statistics and an EBV-DNA level of <100 copies/mL was defined as negative.

### 2.4. Follow-Up

Following the completion of treatment, the patients were followed up once every two months in the first year and once every three months in the second year. Follow-up studies included history recording, detailed physical examination, determination of plasma EBV-DNA copy number, electronic nasopharyngoscopy, MRI examination of the nasopharynx and neck, chest and abdominal CT once every six months, and systemic bone scintigraphy for clinical suspicion of bone metastases. Follow-up was mainly carried out by appointment and telephone calls. The duration of follow-up was calculated as the end of treatment to the last follow-up date. Evaluation indicators included overall survival (OS) and disease-free survival (DFS).

### 2.5. Statistical Analysis

The statistical package for social sciences, version 17.0 software (SPSS 17.0, Chicago, USA) was used for statistical analysis. Bivariate correlation was used to analyze the relationship between pretreatment plasma EBV-DNA levels and tumor burden. The Kaplan-Meier method was used to estimate the disease-free survival (DFS) and overall survival (OS). The log-rank test was used to compare the difference in survival curves. The association between OS or DFS and plasma EBV-DNA levels was assessed by the Pearson Chi-square test. All statistical tests were two-sided, and *P* values < 0.05 were considered statistically significant.

## 3. Results

### 3.1. Correlation between Pretreatment EBV-DNA and Prognosis

**Figure 1:**
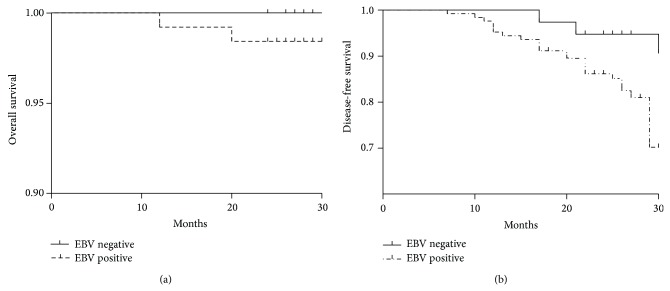
A comparison of overall survival (a) and disease-free survival (b) between pretreatment plasma EBV-DNA negative and positive patients.

The 165 patients were followed up for two years, and the OS rate was 98.8% and DFS rate was 84.2%. For 38 patients with negative pretreatment plasma EBV-DNA levels, the 2-year OS and DFS rates were 100% and 94.4%, respectively. In the remaining 127 patients with positive pretreatment plasma EBV-DNA, 26 patients had distant metastasis and two patients died, resulting in 2-year OS and DFS rates of 98.4% and 80.8%, respectively. There was no significant difference in the 2-year OS between pretreatment plasma EBV-DNA level positive and negative patients (*P* = 1.000); however, the 2-year DFS in EBV-DNA positive patients was lower than in EBV-DNA negative patients (*P* = 0.044) (Figure 1).

### 3.2. Correlation between Pretreatment EBV-DNA and Tumor Burden

The medians of the GTVnx, GTVnd, and total GTV in all patients before treatment were 72.46, 23.26, and 106.25 cm^3^, respectively. The median plasma EBV-DNA copy number was 3790 copies/mL. The pretreatment plasma EBV-DNA levels were significantly correlated with the volume of neck lymph node metastasis and the total GTV (*R* ^2^ = 0.246, 0.213; *P* < 0.001) but not significantly correlated with the GTVnx (*R* ^2^ = 0.051, *P* > 0.05).

## 4. Discussion

Nasopharyngeal carcinoma (NPC) is a malignant tumor with a high incidence. Clinical stage is the major factor affecting the prognosis of NPC, and local recurrence or distant metastasis is the main cause of treatment failure in patients with this disease [7]. Plasma EBV-DNA, as a tumor marker of NPC, has been extensively studied and its reliability has been validated [8–10]. Previous studies found that EBV-DNA could be detected in the plasma of most patients with NPC and proposed that the clinical stage was positively correlated with the EBV-DNA copy number, indicating that the clinical staging of NPC can be complemented at the molecular level [11, 12]. In this study, which included 127 patients with advanced NPC and positive pretreatment plasma EBV-DNA, the median plasma EBV-DNA copy number was 7590 copies/mL for stage III patients, 11 400 copies/mL for stage IVa, and 63 400 copies/mL for stage IVb, which was consistent with previous reports.

It was previously reported that the volume of the nasopharyngeal tumor is a good predictor of prognosis [4, 13]. Tumor volume is the most direct and objective indicator of tumor burden, and the larger the tumor volume, the poorer the prognosis and the lower the OS. Lo et al. considered that the plasma EBV-DNA level may be related to the burden of tumor cells in patients, which may be derived from necrotic tumor cells [14]. In this study, tumor burden was represented by primary tumor volume and lymph node volume. In the 165 patients with locally advanced NPC, the primary tumor volume, the lymph node volume, and the median total volume before treatment were 72.46, 23.26, and 106.25 cm^3^, respectively. A comparative study found that the pretreatment plasma EBV-DNA copy number was significantly correlated with both the cervical lymph node volume and total volume (*P* < 0.001) but was not significantly correlated with the primary tumor volume (*P* > 0.05). These results showed that the main factor for plasma EBV-DNA copy number in patients with locally advanced NPC was the neck lymph nodes.

In this study, 26 of the 165 patients had distant metastasis, and two patients died during the two years of follow-up. We believe that tumor recurrence and metastasis may be associated with higher levels of pretreatment plasma EBV-DNA. Some research showed that pretreatment plasma EBV-DNA levels can be used to evaluate PFS and OS in patients with NPC and noted that patients with a high plasma EBV-DNA level before treatment had a lower survival rate [15, 16]. In this study, 127 patients with NPC had positive pretreatment plasma EBV-DNA, with a median copy number of 9440 copies/mL, significantly greater than 4000 copies/mL, suggesting a poor prognosis. After treatment, the plasma EBV-DNA levels in these patients decreased to an average of 470 copies/mL, less than the 500 copies/mL as reported by Chan et al. These results indicate that radiotherapy can significantly reduce the plasma EBV-DNA level and that prognosis is associated with plasma EBV-DNA level.

Lo et al. found that the median plasma EBV-DNA level in 10 patients with NPC recurrence after radiotherapy was 32 350 copies/mL; however, in 15 patients who achieved remission, the copy number was 0 copies/mL [17]. Tang et al. followed up 86 NPC cases to observe the detection of distant metastasis by [(18)F] fluorodeoxyglucose positron emission tomography and computed tomography (PET/CT) combined with plasma EBV-DNA levels and found that advanced N stage (odds ratio, 2.689; 95% CI, 1.894–3.818) and pretreatment EBV-DNA level (odds ratio, 3.344; 95% CI, 1.825–6.126) were significant risk factors for distant metastases [18]. In the present study, of the 38 patients with negative pretreatment plasma EBV-DNA, none died at the last follow-up, and only two patients had distant metastasis, and the 2-year OS and DFS rates were 100% and 94.4%, respectively. However, of the 127 patients with positive pretreatment plasma EBV-DNA, 26 had distant metastases, two died, and the 2-year OS and DFS rates were 98.4% and 80.8%, respectively; the 2-year DFS rate was lower than that in patients with negative pretreatment plasma EBV-DNA (*P* = 0.044). Consistent with previous findings, these results further suggest that pretreatment plasma EBV-DNA levels may be a predictor in patients with NPC.

In summary, the pretreatment plasma EBV-DNA copy number in patients with locally advanced NPC was significantly positively correlated with the volume of metastatic cervical lymph nodes, indicating that pretreatment plasma EBV-DNA levels may largely depend on the cervical lymph node burden. Pretreatment plasma EBV-DNA level can be used to predict the prognosis of patients with locally advanced NPC, and patients with negative pretreatment plasma EBV-DNA may have a better prognosis.

## Figures and Tables

**Table 1:** Characteristics of 165 patients with locally advanced nasopharyngeal carcinoma.

Characteristics	Pretreatment EBV-DNA (cases/median copies/mL)		Tumor volume (medium, cm^3^)		
	Negative (38)	Positive (127/9440)	GTVnx	GTVnd	Total
Age (years)					
<50	18	77/7210	73.6	29.7	106.3
≥50	20	50/13850	69.3	24.6	108.6
Sex					
Male	30	92/9665	75.1	25.8	115.4
Female	8	35/8620	68.1	26.2	102.8
UICC stage					
III	24	65/7590	64.8	24.5	87.9
IVa	7	43/11400	108.4	17.1	136.8
IVb	7	19/63400	70.4	53.4	144.1
T stage					
T1	7	8/3945	47.0	24.7	62.3
T2	7	18/6265	51.0	29.3	83.5
T3	16	55/12000	67.9	27.4	104.7
T4	8	46/12650	113.7	20.2	142.3
N stage					
N0-1	5	21/5520	76.3	14.5	92.3
N2	26	84/8855	71.8	25.3	103.6
N3	7	22/47700	65.0	58.8	138.0
